# Prevalence of MUTYH Monoallelic Variants in Patients With Hereditary Cancer Using Multigene Panel Testing

**DOI:** 10.1002/cam4.71231

**Published:** 2025-09-26

**Authors:** Gemma Caliendo, Chiara Della Pepa, Alessia Mignano, Luisa Albanese, Luana Passariello, Anna Cozzolino, Francesca Iengo, Anna Maria Molinari, Laura Pesce, Maria Teresa Vietri

**Affiliations:** ^1^ Unit of Clinical and Molecular Pathology AOU University of Campania “Luigi Vanvitelli” Napoli Italy; ^2^ Department of Precision Medicine University of Campania “Luigi Vanvitelli” Napoli Italy; ^3^ Oncology Unit Vallo Della Lucania‐Agropoli Hospital Salerno Italy

## Abstract

**Background:**

The *MUTYH* gene is involved in DNA repair and is known for MAP (*MUTYH*‐associated polyposis), an autosomal recessive disorder that predisposes individuals to colorectal cancer (CRC), with a lifetime risk ranging from 40% to 90%. Homozygosity or double heterozygosity (DH) for pathogenic variants (PVs) in *MUTYH* causes MAP, but several studies suggest that monoallelic PVs may also increase cancer risk, mainly CRC and breast cancer (BC).

**Methods:**

We analyzed *MUTYH* status in a cohort of 130 patients referred to our familial cancer clinic for suspected hereditary cancer, describing their mutations and clinical features, and comparing the *MUTYH* mutation rate between our cancer cohort and a group of 150 healthy volunteers. We also described the genetic profile and clinical features of probands relatives, when possible.

**Results:**

10% of our cancer patients carried a *MUTYH* PV, while the gene was wild type in all the samples from the control group. The most frequent PVs were c.1187G>A (p.Gly396Asp) and c.536A>G (p.Tyr179cys). We found a double mutation (DM) in 6 patients, with one carrying a DM in MUTYH and the other 5 harboring mutations in *MUTYH* and other cancer susceptibility genes (*CHEK2, BRIP1, MLH1*, and *BRCA1*).

**Conclusions:**

The higher *MUTYH* mutation rate observed in the cancer cohort compared with the control group; cancer recurrence observed in the heterozygous carriers and in both maternal and paternal family branches of patients harboring a DM suggests that *MUTYH* PVs may play a role in cancer predisposition and progression, even when monoallelic.

## 1 Background

The introduction of the new techniques of next generation sequencing (NGS) has dramatically changed the scenario in studying tumor genetics; a huge amount of genetic information became much more accessible and, despite constantly updated datasets, not all the information appears simply to manage. Hereditary cancer syndromes are a hot topic in oncology: such syndromes represent a biological model to understand cancer development and find new potential therapeutic targets [1]; it is relevant to comprehend which environmental factors are implicated and how they interfere with genetics to determine different phenotypes. In clinical practice, early identification of affected patients to improve screening and treatment procedures is undoubtedly one of the most important goals in oncology research.

MUTYH gene is well known for its role in MAP (*MUTYH* associated polyposis), an autosomal recessive disorder, but the significance of the monoallelic Pathogenic Variants (PVs) of such gene is much more controversial. The two most common *MUTYH* gene PVs in Western Europeans and North Americans are c.1187G>A (p.Gly396Asp), also known as G396D and G382D, and c.536A>G (p.Tyr179cys), also referred to as Y179C and Y165C; the effect of such mutations, when present in homozygosity or double heterozygosity, is impaired *MUTYH* activity leading to MAP syndrome with different types of polyposis and cancer [2]. In the last decades, several studies have investigated the possibility of increased cancer risk also in monoallelic PV carriers; the results are largely controversial: some authors asserted that the risk of developing colorectal cancer (CRC) is higher in individuals with a *MUTYH* mutation, even if heterozygous [3, 4, 5, 6], while others disputed such theory [7, 8].

This research reports our data about *MUTYH* mutations, derived from the heredo‐familial cancer clinics at the Vanvitelli University in Naples, Italy, with a special focus on patients who are carriers of monoallelic PVs: we evaluated mutation frequency, type of mutation, clinical features, and family history.

The *Escherichia coli* mutY gene was first identified in 1996; in *E. coli* it was demonstrated to play a crucial role in the bacterial repair system, removing and correcting mutations due to oxidative adducts [9]. Human *MUTYH* acts like its *E. coli* homolog, protecting nuclear DNA against the mutagenic effects of the oxidized bases as part of the BER pathway, which is one of the most important DNA repair pathways. The *MUTYH* gene is located on chromosome 1 (p32.1–p34.3) (seq ref. NM_001128425.1) and consists of 16 exons [10]. Scientific interest around MUTYH grew dramatically when it emerged that mutations in such gene were present in patients diagnosed with AFAP (Attenuated Familial Adenomatous Polyposis) without mutations in the *APC* gene. *MUTYH* mutations are reported in 40% of patients with AFAP, 7%–12% of patients with FAP, and 26% of patients with MRCA (Multiple Colorectal Adenomas) [11, 12, 13]. MAP is an autosomal recessive syndrome with a very high penetrance; the risk of developing CRC by the age of 60 years varies from 43%–60% to 80%–90% in the absence of regular surveillance [4].

Germline variants in *MUTYH* are associated with a distinctive mutational signature in CRC, characterized by an excess of G:C>T:A transversions, particularly affecting cancer driver genes such as *APC* and *KRAS*. These alterations reflect the genomic instability caused by defective BER and represent a molecular hallmark of *MUTYH*‐inactivated tumors [14]. Beyond CRC, increasing evidence suggests that defective *MUTYH* function may also contribute to tumorigenesis in other tissues, including breast, endometrium, and urinary tract, through accumulation of somatic mutations and generalized genomic instability. Several pan‐cancer studies have demonstrated *MUTYH* inactivation or loss of heterozygosity across various tumor types [15], and reduced *MUTYH* expression has been reported in breast and gynecologic tumors from germline variant carriers [16]. Recent findings have led to the proposal of the term *MUTYH*‐associated tumor syndrome (MATS) to more accurately reflect the broad cancer spectrum observed in carriers of germline *MUTYH* mutations, beyond the classical colorectal phenotype of MAP. In this context, monoallelic *MUTYH* variants are frequently identified in patients with early‐onset or familial cancers, and some tumors from these individuals display molecular features similar to those of biallelic cases (e.g., G:C>T:A transversions and signature 36), suggesting a potential pathogenic role, especially in the presence of somatic loss of heterozygosity [17].

## 2 Materials and Methods

### 2.1 Patients

This study was carried out in accordance with the World Medical Association Helsinki Declaration (1964). Informed consent was obtained from all the subjects, and the study was approved and conducted according to the ethical guidelines of the University of Campania “Luigi Vanvitelli” (n.25455‐07/09/2021). All individuals included in our study were of Italian origin and recruited from Campania, a region in southern Italy. The study was conducted at the U.O.C. Clinical and Molecular Pathology‐Heredo‐familiar Cancer, A.O.U. University of Campania “Luigi Vanvitelli.”

We analyzed 130 cases of patients affected by cancer, who were referred to our heredo‐familial cancer clinics for a suspicious heredo‐familiar cancer syndrome: HBOC (Hereditary Breast Ovarian Cancer), Lynch Syndrome, familiar CRC, polyposis. The probands, 41 males and 89 females, received genetic counseling and a pedigree was generated; the age ranged from 19 to 80 years old (age at the time of the test), median age was 50 years old.

### 2.2 Genetic Testing

DNA from blood samples was extracted using the QIAmp DNA Blood Mini Kit (250) according to the manufacturer's instructions.

For mutational analysis, we used two Smart Seq Sequencing Cancer Panels on a MiSeq platform (Illumina, HCS, Sophia Genetics, Switzerland), analyzing the genes which are listed below: *BRCA1* (NM_007295), *BRCA2* (NM_000059), *PALB2* (NM_024675), *ATM* (NM_000051.4), *BARD1* (NM_000465.4), *BRP1* (NM_001003694.2), *CDH1* (NM_004360), *CHEK2* (NM_007194), *NBN* (NM_002485), *PTEN* (NM_000314.8), *RAD51C* (NM_058216), *RAD51D* (NM_002878.3), *STK11* (NM_000455.5), *TP53* (NM 000546), *MUTYH* (NM_001128425.1), *POLE* (NM_006231.2), *POLD1* (NM_001256849.1), *EPCAM* (NM_002354.2), *MSH2* (NM_000251.2), *MSH6* (NM_000179.2), *MLH1* (NM_000249.3), *CTNNB1* (NM_001904.3), *APC* (NM_000038.5), *PMS2* (NM_000535.6).

The results have been interpreted from the Mutation Surveyor software, version 3.24 (Softgenetics, State College, PA, USA); ClinVar, LOVD databases were used for the identification and classification of genetic variants (benign, pathogenic, Variants of Uncertain Significance—VUS) [18].

Sanger sequencing, as previously described [19, 20], was performed to evaluate 150 samples from healthy volunteers, 59 males and 91 females, with age varying from 18 to 82 years old and median age 50 years old. In such control cohort we analyzed the *MUTYH* PVs: c.1187G>A (p.Gly396Asp), c.536A>G (p.Tyr179cys), c.270C>A (p.Tyr90*), c.1437_1439delGGA (p.Glu480del).

### 2.3 Segregation Analysis

To proceed with segregation and co‐segregation analysis, sanger sequencing was also used to test our probands familiars. When feasible, parents underwent sanger sequencing for the *MUTYH* PV observed in their descendants; for patients with a Double Mutation (DM), consisting of a *MUTYH* mutation together with a mutation in another susceptibility gene, both mutations were checked.

### 2.4 In Silico Analysis

For patients carrying a variant in *MUTYH* but also a VUS or a novel variant in another cancer susceptibility gene, we performed in silico analysis. We used, via Ensembl Variant Effect Predictor (VEP) (https://www.ensembl.org/Tools/VEP, accessed on 10 June 2025), Polymorphism Phenotyping v2 (PolyPhen‐2), SIFT, Rare Exome Variant Ensemble Learner (REVEL), and BayesDel, to predict the effect of missense variants; while intronic and 3′UTR variants were analyzed using CADD (https://cadd.gs.washington.edu/, accessed on 6 May 2025) and SpliceAI (https://spliceailookup.broadinstitute.org/, accessed on 6 May 2025). Variants were classified according to ACMG/VCEP guidelines (https://clinicalgenome.org) into five categories: pathogenic (class I), likely pathogenic (II), uncertain significance (III), likely benign (IV), and benign (V) [21].

These tools have been largely validated in previous studies: PolyPhen 2 evaluates the potential effects of each aminoacid substitution on the structural and functional properties of the protein by applying physical and comparative approaches. It provides three possible output whether probably damaging (values are more rapidly to one), possibly damaging or benign (values are varieties from 0 to 0.95) [22]. SIFT works similarly, providing a score ranging from 0 to 1: scores ≤ 0.05 are predicted by the algorithm to be damaging amino acid substitutions, whereas scores > 0.05 are considered to be tolerated [23]. REVEL provides a continuous score ranging from 0 to 1, where higher values indicate a greater likelihood of pathogenicity; the commonly adopted threshold is ≥ 0.5. BayesDel is based on a Bayesian model that incorporates evolutionary conservation and allele frequency data, generating a score between −1 and 1, with values > 0.16 suggestive of a pathogenic effect. Combined Annotation Dependent Depletion, CADD, calculates the score combining different parameters derived from surrounding sequence context, gene model annotations, evolutionary constraint, epigenetic measurements and functional predictions. For any given variant, all of these annotations are integrated into a single CADD score that ranges from 1 to 99, higher values indicating more deleterious cases [24]. SpliceAI calculates four Δ scores: acceptor gain (AG), acceptor loss (AL), donor gain (DG), and donor loss (DL). These scores measure the impact of the variant on the splicing site. The Δ score ranges from 0 to 1, with higher values indicating a greater probability that the variant affects splicing. Variants with a Δ score below 0.20 are generally considered unlikely to have a significant impact on splicing, while those with Δ scores above 0.80 are typically associated with a strong impact on splicing [25]. VEP, developed by the Ensembl Project, is a variant annotation tool that provides functional, genomic, and predictive information by integrating metapredictors such as PolyPhen‐2, SIFT, REVEL, BayesDel, CADD, SpliceAI and offering standardized outputs useful for clinical classification [26].

## 3 Results

### 3.1 MUTYH Mutation Rate and Type of Mutation

We found a *MUTYH* mutation in 13 probands out of 130 (10%) in our cancer patients' cohort, while we did not find any M*UTYH* mutation in the control group, composed of 150 samples from sane volunteers.

Table 1 shows genotypes and clinical features of the 13 mutated patients.

**TABLE 1:** Probands with mutations in MUTYH: Mutations, VUS, or novel variants in other genes, clinical phenotype, and family history.

ID	M/F	Mutation in *MUTYH*	dbSNP ID	Mutation in other susceptibility genes	VUS/novel variants in other genes	Clinical phenotype and age at diagnosis	Paternal family history	Maternal family history
Patient 1	F	c.270C>A (p.Tyr90*) c.1187G>A (p.Gly396Asp)	rs121908380 rs36053993	None	None	MAP and CRC 48	c.270C>A (p.Tyr90Ter) Father, heterozygous carrier, sane; LC in paternal uncle	c.1187G>A (p.Gly396Asp) Mother, heterozygous carrier, sane; CRC in maternal grandmother, CRC in maternal aunt
Patient 2	M	c.1187G>A (p.Gly396Asp) HOMOZYGOUS	rs36053993	None	None	GC 44, CRC 48	c.1187G>A (p.Gly396Asp) Father, heterozygous carrier, affected from PC and CRC; paternal aunt with BC; one paternal aunts with CRC, PaC, LC, and PC	c.1187G>A (p.Gly396Asp) Mother, heterozygous carrier, sane; maternal aunts with RCC and CRC
Patient 3	F	c.536A>G (p.Tyr179cys)	rs34612342	***CHEK2**:* c.470T>C (p.Ile157Thr)	None	BBC 47	*CHEK2* WT, *MUTYH*: c.536A>G (p.Tyr179cys) Father sane; BC in paternal grandmother	No genetic data, mother with PaC; CRC in maternal grandmother and maternal grandfather with RCC
Patient 4	F	c.1187G>A (p.Gly396Asp)	rs36053993	***BRIP1**:* c.55dup (p.Tyr19fs*2)	None	BC 53	No genetic data, father with LC, two paternal aunts with LC and one paternal aunt with CRC	*BRIP1* WT, *MUTYH* WT Mother sane; two maternal aunts with BC
Patient 5	M	c.536A>G (p.Tyr179cys)	rs34612342	***MLH1**:* c.304G>A (p.Glu102Lys)	***PMS2**: Novel* c.*146C>A	Polyposis 19	No genetic data, father dead from CRC at 31; paternal aunts with CRC, UT; paternal grandmother with EC and paternal grandfather with PC and CRC and one aunt with EC and one aunt with CRC	*MLH1* WT, *PMS2* WT, *MUTYH:* c.536A>G (p.Tyr179cys) Mother, heterozygous carrier, sane; two maternal aunts with CRC; maternal grandmother with BC and TC
Patient 6	M	c.1187G>A (p.Gly396Asp)	rs36053993	***BRCA1**:* c.3756_3759del (p.Ser1253Argfs*10)	None	CRC 57	No genetic data, no clinical data	No genetic data, mother affected from BC and EC; maternal aunt with BC
Patient 7	F	c.1187G>A (p.Gly396Asp)	rs36053993	***BRCA1**:* c.4964_4982del (p.Ser1655Tyrfs*16)	None	BC 35, sarcoma 11	No genetic data, no clinical data	No genetic data, no clinical data
Patient 8	F	c.536A>G (p.Tyr179cys)	rs34612342	None	***RAD51D**: Novel* c.145‐35A>G ***PMS2***: *Novel* c.*110del	BC 60	No genetic data, father dead from CRC; paternal cousin with OC	No genetic data, no clinical data; maternal cousin with BC
Patient 9	M	c.1187G>A (p.Gly396Asp)	rs36053993	None	***APC**: VUS* c.7610C>G (P.Ser2537Cys) rs758106362; ***APC** Novel* c.221‐43T>C ***CHEK2**: Novel* c.684‐86A>G	Melanoma 23, TC 12	*MUTYH:* c.1187G>A (p.Gly396Asp) Father sane, paternal aunt with EC	*MUTYH* WT, mother sane, maternal grandfather with CRC; maternal grandmother with EC and RCC; three maternal aunts with EC
Patient 10	F	c.1437_1439delGGA (p.Glu480del)	rs587778541	None	None	CRC 53	No genetic data, father with PC	c.1437_1439delGGA (p.Glu480del) Mother with BC and CRC
Patient 11	F	c.1187G>A (p.Gly396Asp)	rs36053993	None	None	BC 49, OC 54	No genetic data, paternal aunt with GC	No genetic data, mother with biliary tract cancer, maternal aunt with BC and two cousins with LC and one cousin with BC
Patient 12	M	c.1187G>A (p.Gly396Asp)	rs36053993	None	None	Polyposis, CRC 68	No genetic data, father with GC	No genetic data, no clinical data
Patient 13	F	c.536A>G (p.Tyr179cys)	rs34612342	None	None	MSI and EC 66	No genetic data, no clinical data Cousin with PaC	No genetic data, no clinical data Cousin with PaC

Abbreviations: BBC, Bilateral breast cancer; BC, Breast cancer; CRC, Colorectal cancer; EC, Endometrial cancer; GC, Gastric cancer; LC, Lung cancer; MAP, MUTYH‐associated polyposis; OC, Ovarian cancer; PaC, Pancreas cancer; PC, Prostate cancer; RCC, Renal cell cancer; TC, Thyroid cancer.

In particular, 2 patients out of 130 (1.5%) had a biallelic mutation: patient 1 had a Double Heterozygosity (DH) in *MUTYH* consisting of the PV c.270C>A (p.Tyr90*) and the PV c.1187G>A (p.Gly396Asp); patient 2 had a homozygous genotype for the PV c.1187G>A (p.Gly396Asp).

11 patients out of 130 (8.5%) had a monoallelic PV in *MUTYH*: 6 out of 11 patients harbored the PV c.1187G>A (p.Gly396Asp); 4 out of 11 carried the PV c.536A>G (p.Tyr179cys); 1 out of 11 had the PV c.1437_1439delGGA (p.Glu480del).

No VUS and/or novel variants of the *MUTYH* gene were found.

In our series the prevalence rates of the PVs c.1187G>A (p.Gly396Asp) and c.536A>G (p.Tyr179cys) were 6.2% (8/130) and 3% (4/130) respectively, resulting in a cumulative prevalence rate of 9.2% (12/130).

Among the group of the 13 *MUTYH*‐mutated patients, 62% (8/13) presented the PV c.1187G>A (p.Gly396Asp); 31% (4/13) cried the PV c.536A>G (p.Tyr179cys); 7.7% (1/13) the mutation c.1437_1439delGGA (p.Glu480del) and 7.7% (1/13) the mutation c.270C>A (p.Tyr90*).

5 patients out of 130 (4.6%) presented a double mutation (DM), consisting of a pathogenic mutation in other susceptibility genes together with a *MUTYH* PV: such mutations involved *BRCA1* in two cases and *CHEK2, BRIP1* and *MLH1* in the other three.

Three patients out of 130 (2.3%) had a novel variant and/or a VUS in other genes, these were *PMS2, RAD51, APC* and *CHEK2*.

### 3.2 In Silico Analysis

Table 2 shows the results of the in silico analysis. Among the 13 patients harboring a *MUTYH* variant there were 3 patients carrying a VUS and/or a novel variant in other cancer susceptibility genes, in particular we found one VUS: the VUS in *APC*, c.7610C>G (P.Ser2537Cys), seen in patient 9, and 5 novel variants. The 5 novel variants were the intronic *APC* variant, c.221‐43T>C, and the intronic CHEK2 variant, c.684‐86A>G, observed in patient 9; the intronic *RAD51D* variant, c.145‐35A>G, and the 3′UTR *PMS2* variant, C.*110del, seen in patient 8; the 3′UTR *PMS2* variant, c.*146C>A, found in patient 5.

**TABLE 2:** In silico analysis.

Gene	Variant	Variant type	Description in ClinVar	Tool	Score	Prediction	Variant classification according to ACMG/VCEP criteria
*APC*	c.7610C>G (p.Ser2537Cys)	Missense	VUS	SIFT	0	Deleterious low confidence	Likely pathogenic (II) PP3
				PolyPhen‐2	0.998	Probably damaging	
				BayasDel	0.262	Probably damaging	
				Revel	0.550	Deleterious low confidence	
*APC*	c.221‐43T>C	Intronic	Novel	CADD	1.536	No deleterious	Likely benign (IV) BP4
				SpliceAI	Donor gain (0.01) Acceptor gain (0.00)	No impact on splicing	
*CHEK2*	c.684‐86A>G	Intronic	Novel	CADD	4.117	No deleterious	Likely benign (IV) BP4
				SpliceAI	Donor gain (0.39) Acceptor gain (0.71)	Possible impact on splicing	
*RAD51D*	c.145‐35A>G	Intronic	Novel	CADD	0.019	No deleterious	Likely benign (IV) BP4
				SpliceAI	Donor gain (0.01) Acceptor gain (0.01)	No impact on splicing	
*PMS2*	c.*110del	UTR	Novel	CADD	0.724	No deleterious	Likely benign (IV) BP4
				SpliceAI	Donor gain (0.05) Acceptor gain (0.00)	No impact on splicing	
*PMS2*	c.*146C>A	UTR	Novel	CADD	6.279	No deleterious	Likely benign (IV) BP4
				SpliceAI	Donor gain (0.05) Acceptor gain (0.20)	No impact on splicing	
*MUTYH*	c.1187G>A (p.Gly396Asp)	Missense	Pathogenic	SIFT	0	Deleterious	Likely pathogenic (II) PP3
				PolyPhen‐2	0.991	Probably damaging	
				BayasDel	0.210	Probably damaging	
				Revel	0.954	Deleterious low confidence	
*MUTYH*	c.536A>G (p.Tyr179cys)	Missense	Pathogenic	SIFT	0	Deleterious	Likely pathogenic (II) PP3
				PolyPhen‐2	1	Probably damaging	
				BayasDel	0.144	Probably damaging	
				Revel	0.963	Deleterious low confidence	

The results of the bioinformatic prediction tools were concordant in classifying the VUS in *APC* as likely pathogenic (Class II, PP3); all the novel variants as likely benign (Class IV, BP4) or benign (Class V, BP4); the two most frequent *MUTYH* variants, c.1187G>A (p.Gly396Asp) and c.536A>G (p.Tyr179Cys), as likely pathogenic (Class II, PP3).

### 3.3 Clinical Features and Family History

The clinical phenotype and family history of the 13 probands harboring a mutation in *MUTYH* are described in Table 1. Most of them, 6 out of 13, were referred to our Clinic for a suspicious heredo‐familial CRC with or without polyposis; 5 had BC; 1 had melanoma and thyroid cancer; 1 had endometrial cancer with high microsatellite instability (MSI). CRC cases and/or polyposis were noted in the pedigree of 7 patients out of 13.

**FIGURE 1:**
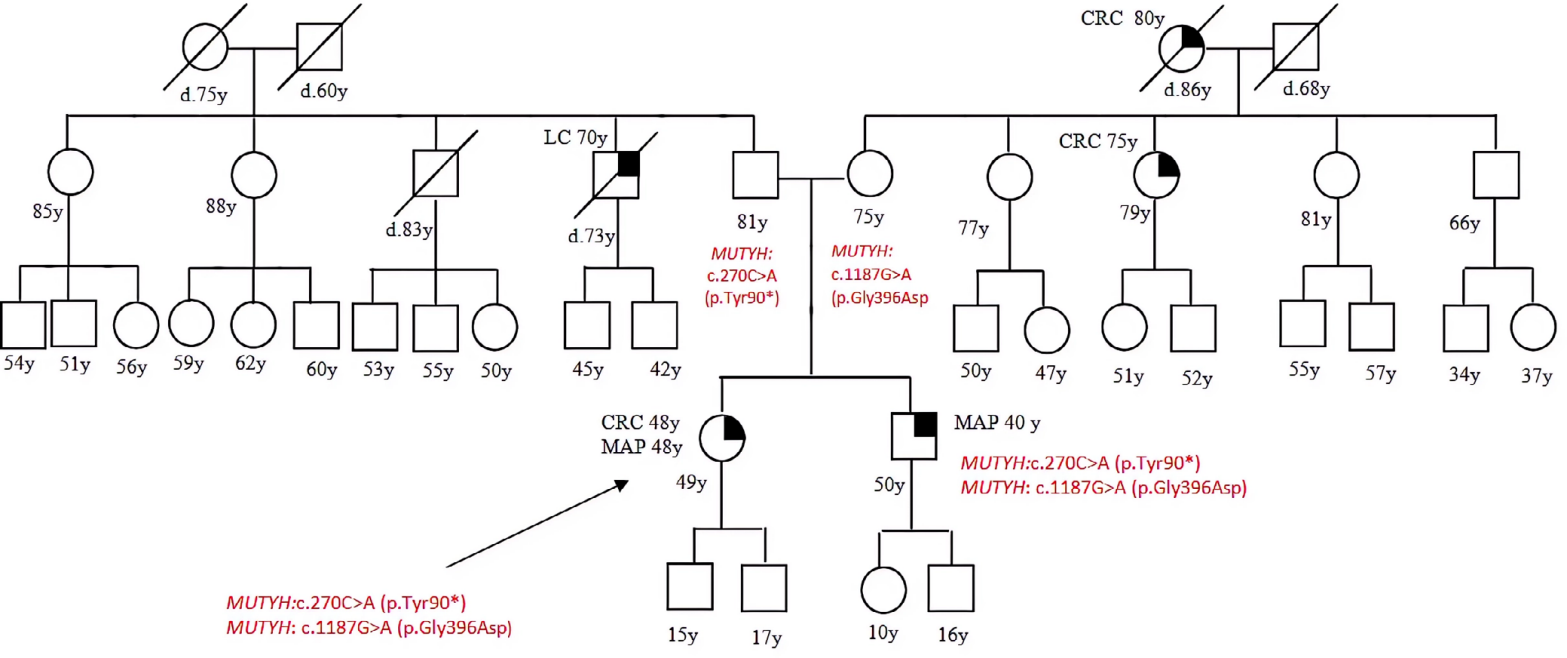
Pedigree of the proband carrying a DH in *MUTYH* c.270C>A (p.Tyr90*) and c.1187G>A (p.Gly396Asp). CRC, Colorectal cancer; MAP, MUTYH‐associated polyposis; OC, Ovarian cancer; BC, Breast cancer; BBC, Bilateral breast cancer; PC, Prostate cancer; LC, Lung cancer; RCC, Renal cell cancer; GC, Gastric cancer; EC, Endometrial cancer; PaC, Pancreas cancer; TC, Thyroid cancer; TeC, Testicular cancer.

Patient 1 presented with polyposis and CRC at 48 years old, she was referred to our clinic for genetic testing after diagnosis of MAP in her brother. She was found to harbor a DH (Double Heterozygosity) in *MUTYH*: she inherited the *MUTYH* pathogenic mutation c.270C>A (p.Tyr90*) from her father and the c.1187G>A (p.Gly396Asp) mutation from her mother. Her brother, who had the same DH, was diagnosed with MAP at the age of 40; their parents, who were both carriers of a monoallelic mutation, were both sane at the age of 81 and 75 (Figure 1).

**FIGURE 2:**
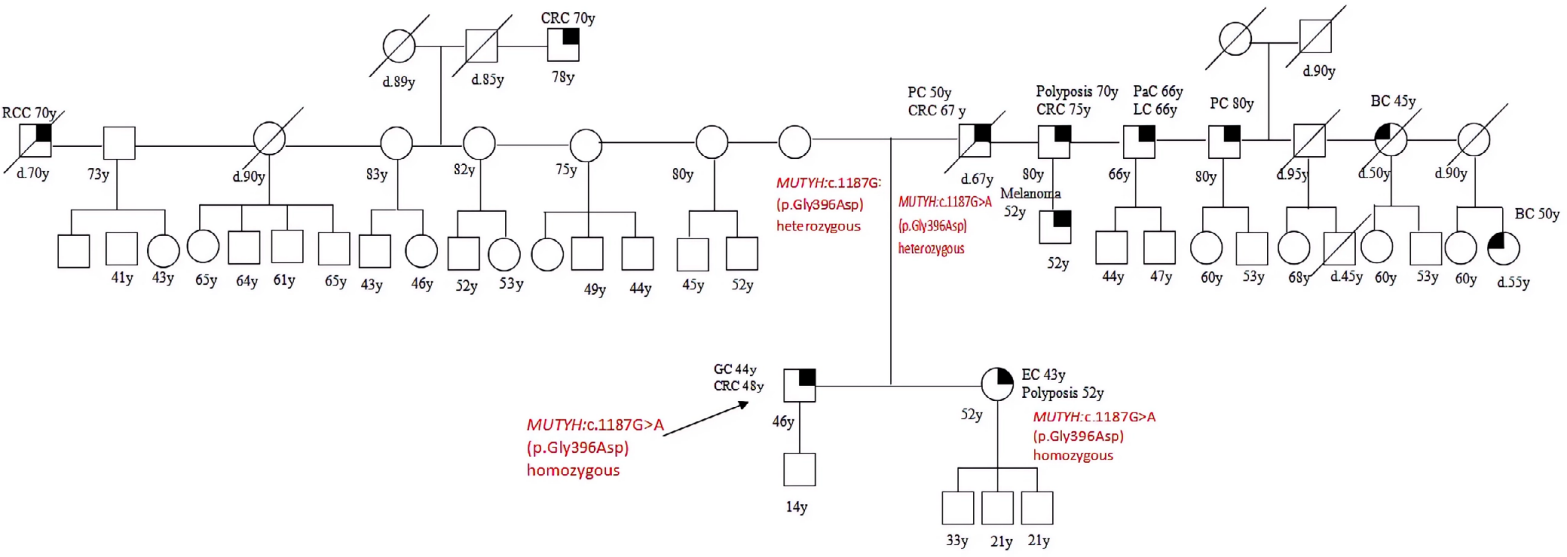
Pedigree of the proband carrying a DM in *MUTYH*, homozygous c.1187G>A (p.Gly396Asp). CRC, Colorectal cancer; MAP, MUTYH‐associated polyposis; OC, Ovarian cancer; BC, Breast cancer; BBC, Bilateral breast cancer; PC, Prostate cancer; LC, Lung cancer; RCC, Renal cell cancer; GC, Gastric cancer; EC, Endometrial cancer; PaC, Pancreas cancer; TC, Thyroid cancer; TeC, Testicular cancer.

Patient 2 was diagnosed with gastric cancer at 44 and CRC at 48, he carried a homozygous *MUTYH* mutation, c.1187G>A (p.Gly396Asp); segregation analysis showed that his parents were both heterozygous carriers of such mutation, his father suffered from PC and CRC, his mother was sane; his sister, who had OC at 43, had the equal homozygous mutation; his son was a heterozygous carrier and healthy at the time of the test (Figure 2).

**FIGURE 3:**
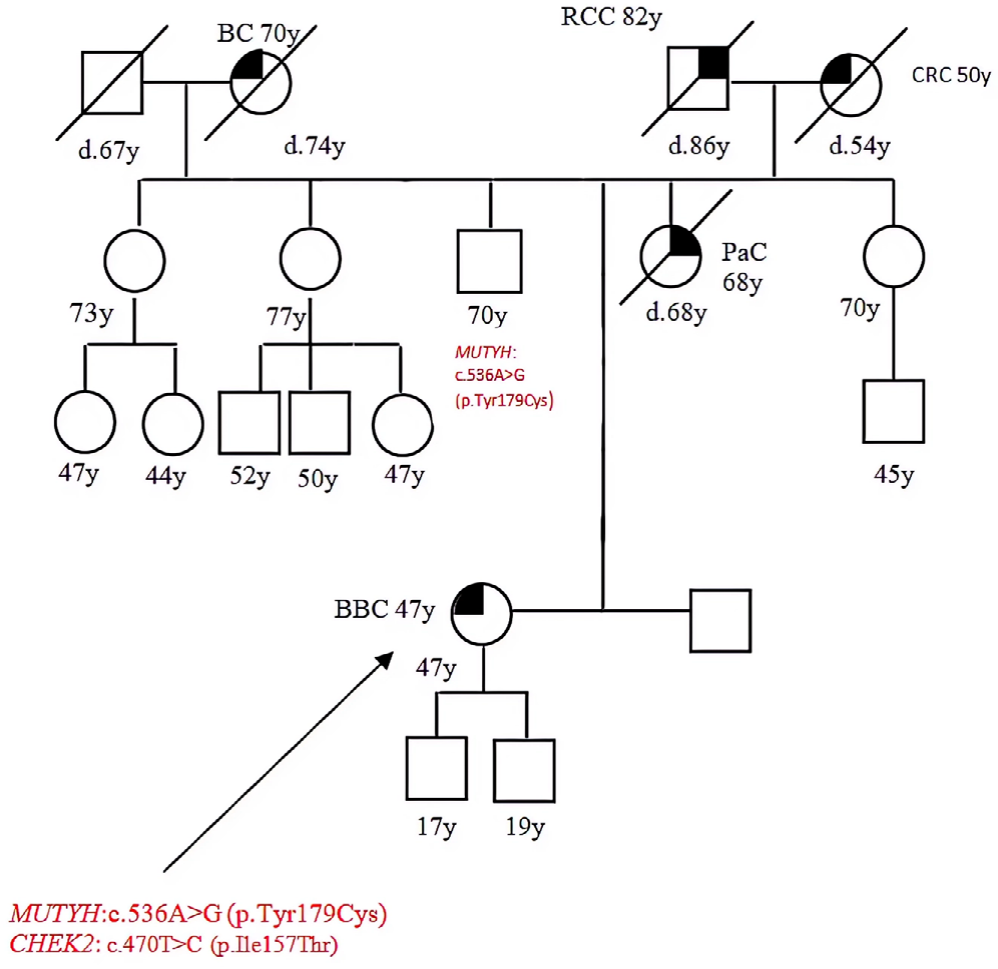
Pedigree of the proband carrying a DM, *MUTYH* mutation c.536A>G (p.Tyr179cys) and *CHEK2* mutation c.470T>C (p.Ile157Thr). CRC, Colorectal cancer; MAP, MUTYH‐associated polyposis; OC, Ovarian cancer; BC, Breast cancer; BBC, Bilateral breast cancer; PC, Prostate cancer; LC, Lung cancer; RCC, Renal cell cancer; GC, Gastric cancer; EC, Endometrial cancer; PaC, Pancreas cancer; TC, Thyroid cancer; TeC, Testicular cancer.

Patient 3 presented with bilateral BC at 47, her mother died from pancreas cancer, her maternal grandmother had CRC, her paternal grandmother had BC; she had the *MUTYH* c.536A>G (p.Tyr179cys), but also a mutation in *CHEK2:* c.470T>C (p.Ile157Thr) that is known as pathogenic. Segregation analysis showed that she inherited the *MUTYH* mutation from her father and the *CHEK2* mutation from her mother (Figure 3).

**FIGURE 4:**
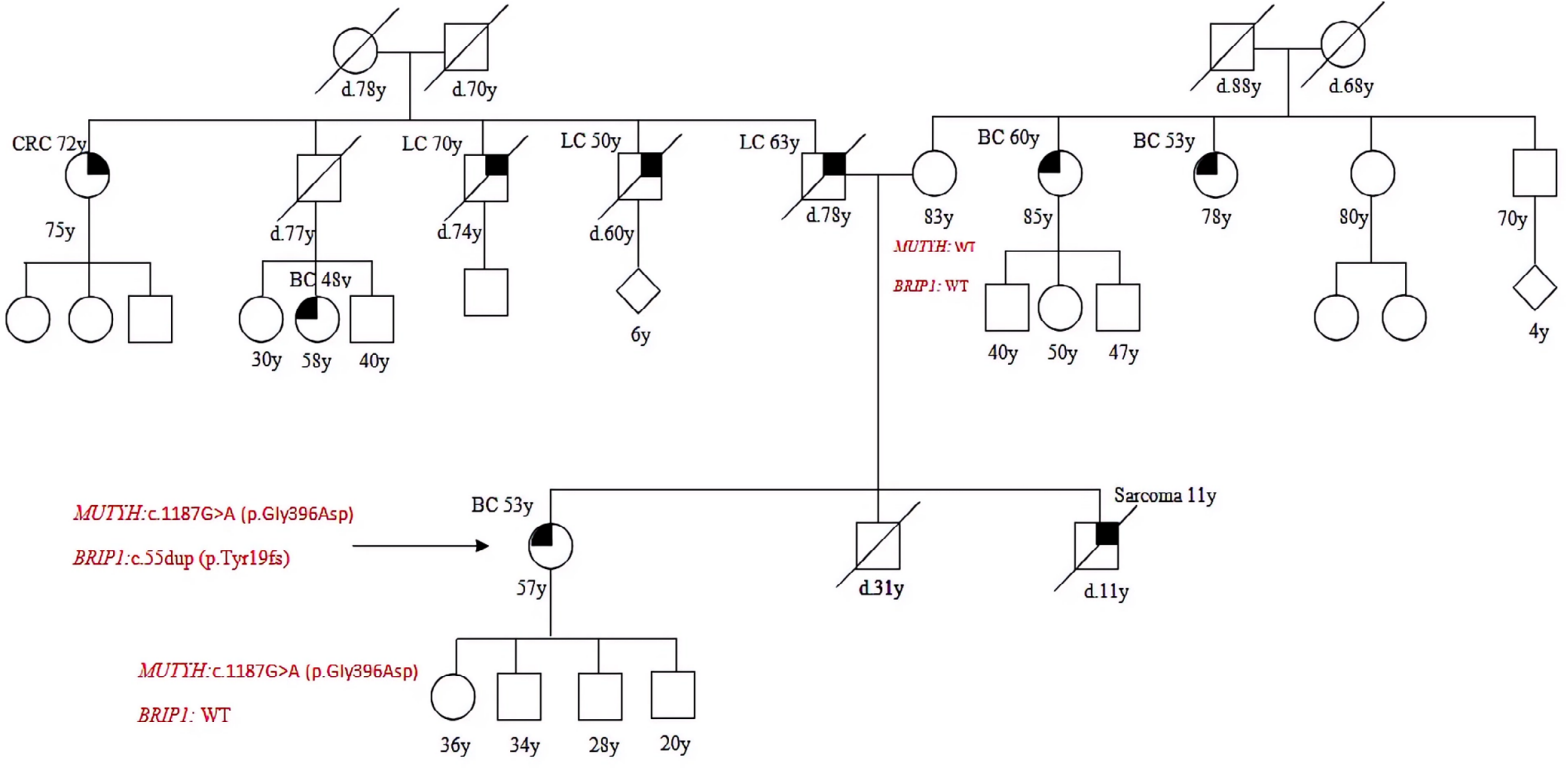
Pedigree of the proband carrying a DM: *MUTYH* mutation c.1187G>A (p.Gly396Asp) and *BRIP1* mutation: C.55dup (p.Tyr19fs*2). CRC, Colorectal cancer; MAP, MUTYH‐associated polyposis; OC, Ovarian cancer; BC, Breast cancer; BBC, Bilateral breast cancer; PC, Prostate cancer; LC, Lung cancer; RCC, Renal cell cancer; GC, Gastric cancer; EC, Endometrial cancer; PaC, Pancreas cancer; TC, Thyroid cancer; TeC, Testicular cancer.

Patient 4 was affected by BC at 53 years old and she reported multiple cases of lung cancer and one case of sarcoma in her family history. She had the *MUTYH* mutation, c.1187G>A (p.Gly396Asp), but also a pathogenic mutation in *BRIP1*, c.55dup (p.Tyr19fs*2). We also studied her mother who was Wild Type (WT) for both *MUTYH* and *BRIP1* and her daughter who inherited the *MUTYH* mutation only (Figure 4).

**FIGURE 5:**
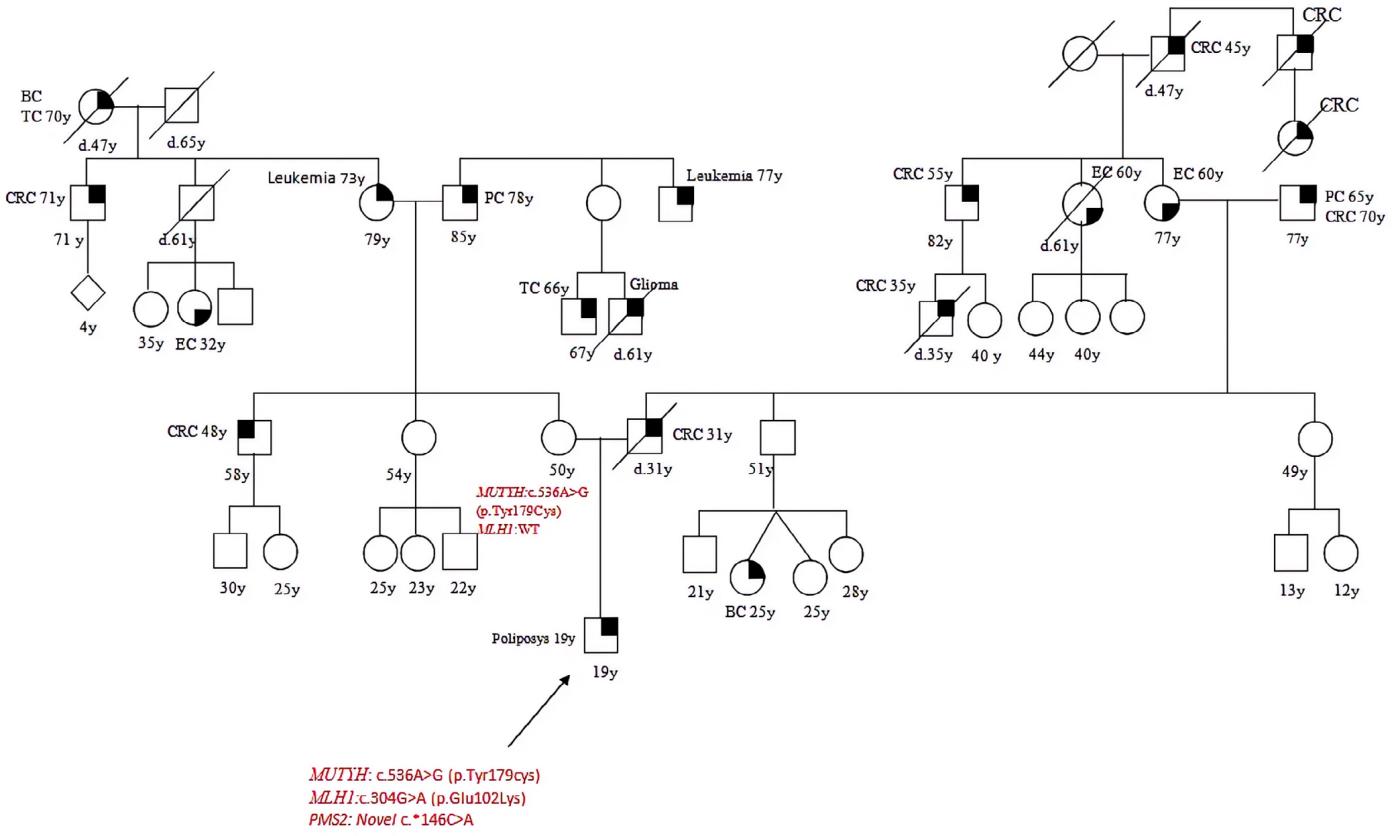
Pedigree of the proband carrying a DM: *MUTYH* mutation c.536A>G (p.Tyr179cys) and *MLH1* mutation c.304G>A (p.Glu102Lys). CRC, Colorectal cancer; MAP, MUTYH‐associated polyposis; OC, Ovarian cancer; BC, Breast cancer; BBC, Bilateral breast cancer; PC, Prostate cancer; LC, Lung cancer; RCC, Renal cell cancer; GC, Gastric cancer; EC, Endometrial cancer; PaC, Pancreas cancer; TC, Thyroid cancer; TeC, Testicular cancer.

Patient 5 was diagnosed with polyposis at 19 years old and his father died from CRC at 31. He harbored the *MUTYH* mutation c.536A>G (p.Tyr179cys) and the pathogenic mutation in *MLH1*, c.304G>A (p.Glu102Lys). In this proband we also identified a novel variant in *PMS2*: c.*146C>A, that we describe for the first time and was likely benign (class IV, BP4) at the in silico analysis (Table 2). Genetic testing on patient's mother showed that she was wild type for *MLH1* and *PMS2* but carried the same *MUTYH* PV (Figure 5).

**FIGURE 6:**
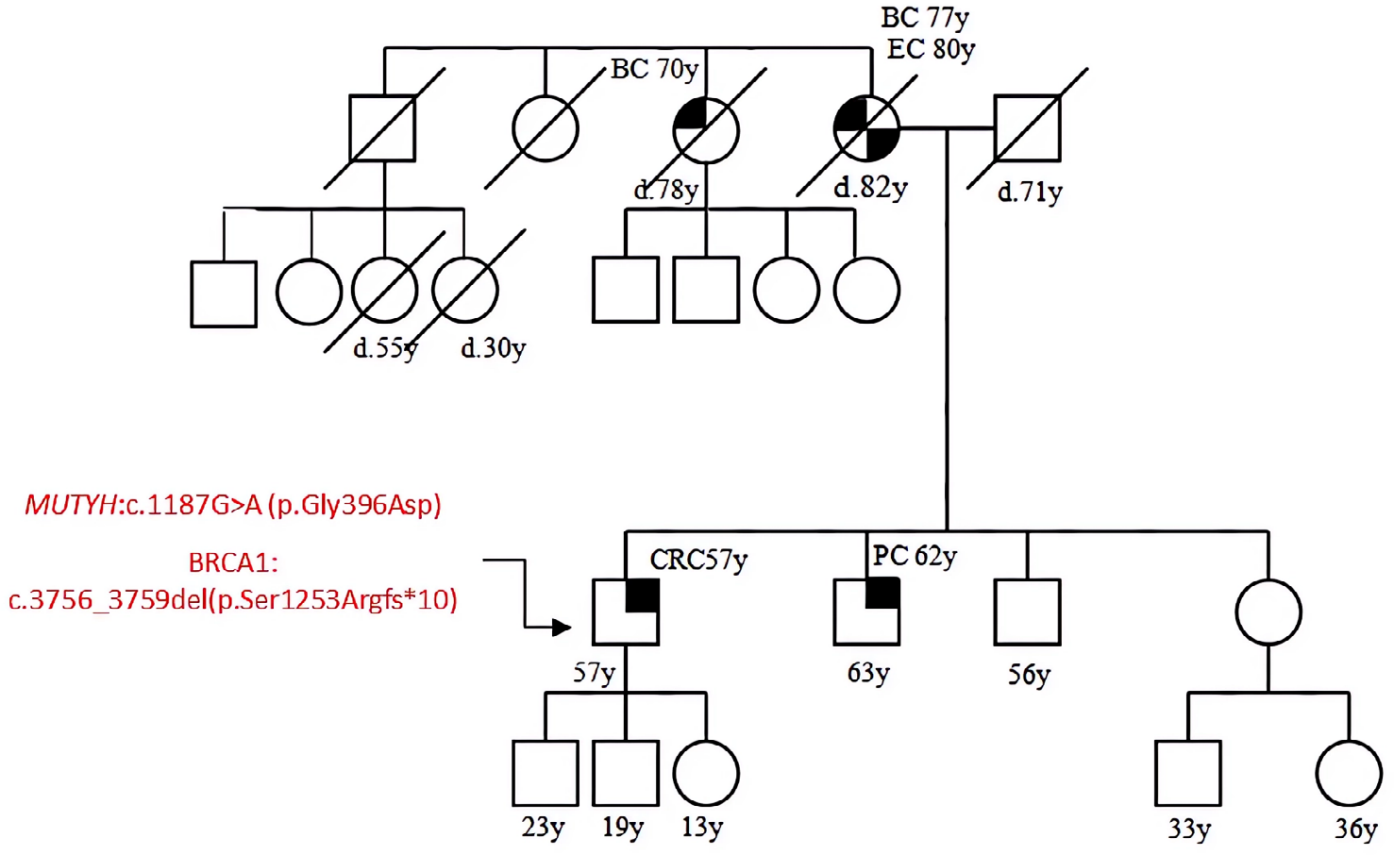
Pedigree of the proband carrying a DM: *MUTYH* mutation c.1187G>A (p.Gly396Asp) and *BRCA1* c.3756_3759del (p.Ser1253Argfs*10). CRC, Colorectal cancer; MAP, MUTYH‐associated polyposis; OC, Ovarian cancer; BC, Breast cancer; BBC, Bilateral breast cancer; PC, Prostate cancer; LC, Lung cancer; RCC, Renal cell cancer; GC, Gastric cancer; EC, Endometrial cancer; PaC, Pancreas cancer; TC, Thyroid cancer; TeC, Testicular cancer.

Patient 6 presented with CRC at 57; investigating his family history it was noted that one of his brothers had prostate cancer, his mother had BC and endometrial cancer, a maternal aunt died from BC. This patient had the *MUTYH* mutation c.1187G>A (p.Gly396Asp) but also a mutation, known to be pathogenic, in *BRCA1*: c.3756.3759del (p.Ser1253Argfs*10). For this patient it was not possible to extend genetic test to familiars (Figure 6).

**FIGURE 7:**
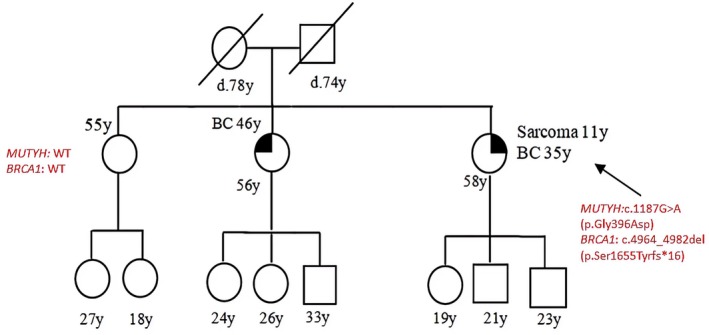
Pedigree of the proband carrying a DM: *MUTYH* mutation c.1187G>A (p.Gly396Asp) and *BRCA1* mutation c.4964_4982del (p.Ser1655Tyrfs*16). CRC, Colorectal cancer; MAP, MUTYH‐associated polyposis; OC, Ovarian cancer; BC, Breast cancer; BBC, Bilateral breast cancer; PC, Prostate cancer; LC, Lung cancer; RCC, Renal cell cancer; GC, Gastric cancer; EC, Endometrial cancer; PaC, Pancreas cancer; TC, Thyroid cancer; TeC, Testicular cancer.

Patient 7 was affected from sarcoma at 11 years old and from BC at 35. She presented the *MUTYH* mutation, c.1187G>A (p.Gly396Asp), and a pathogenic mutation in *BRCA1*, c.4964_4982del (p.Ser1655Tyrfs*16). She had two sisters: one underwent genetic analysis and was sane at the time of the counseling and WT for both genes; the other suffered from BC but was not genetically studied. Segregation analysis was not feasible as parents were deceased (Figure 7).

**FIGURE 8:**
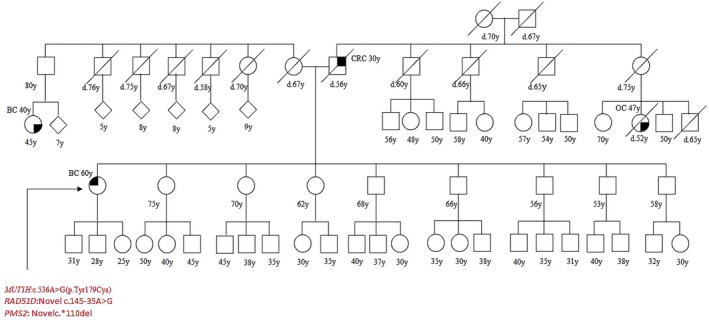
Pedigree of the proband carrying the monoallelic *MUTYH* mutation c.536A>G (p.Tyr179cys) and novel variants in *PMS2* and *RAD51*. CRC, Colorectal cancer; MAP, MUTYH‐associated polyposis; OC, Ovarian cancer; BC, Breast cancer; BBC, Bilateral breast cancer; PC, Prostate cancer; LC, Lung cancer; RCC, Renal cell cancer; GC, Gastric cancer; EC, Endometrial cancer; PaC, Pancreas cancer; TC, Thyroid cancer; TeC, Testicular cancer.

Patient 8 had BC at 60 and her father died from CRC at 56. She harbored the c.536A>G (p.Tyr179cys) PV in *MUTYH* and two novel variants: (i) *RAD51D*: c.145‐35A>G; (ii) *PMS2*: c.*110del, both them have been detected and described for the first time at our laboratory; both them resulted benign (class V, BP4) at the bioinformatic prediction (Table 2). It was not possible to test patients parents (Figure 8).

**FIGURE 9:**
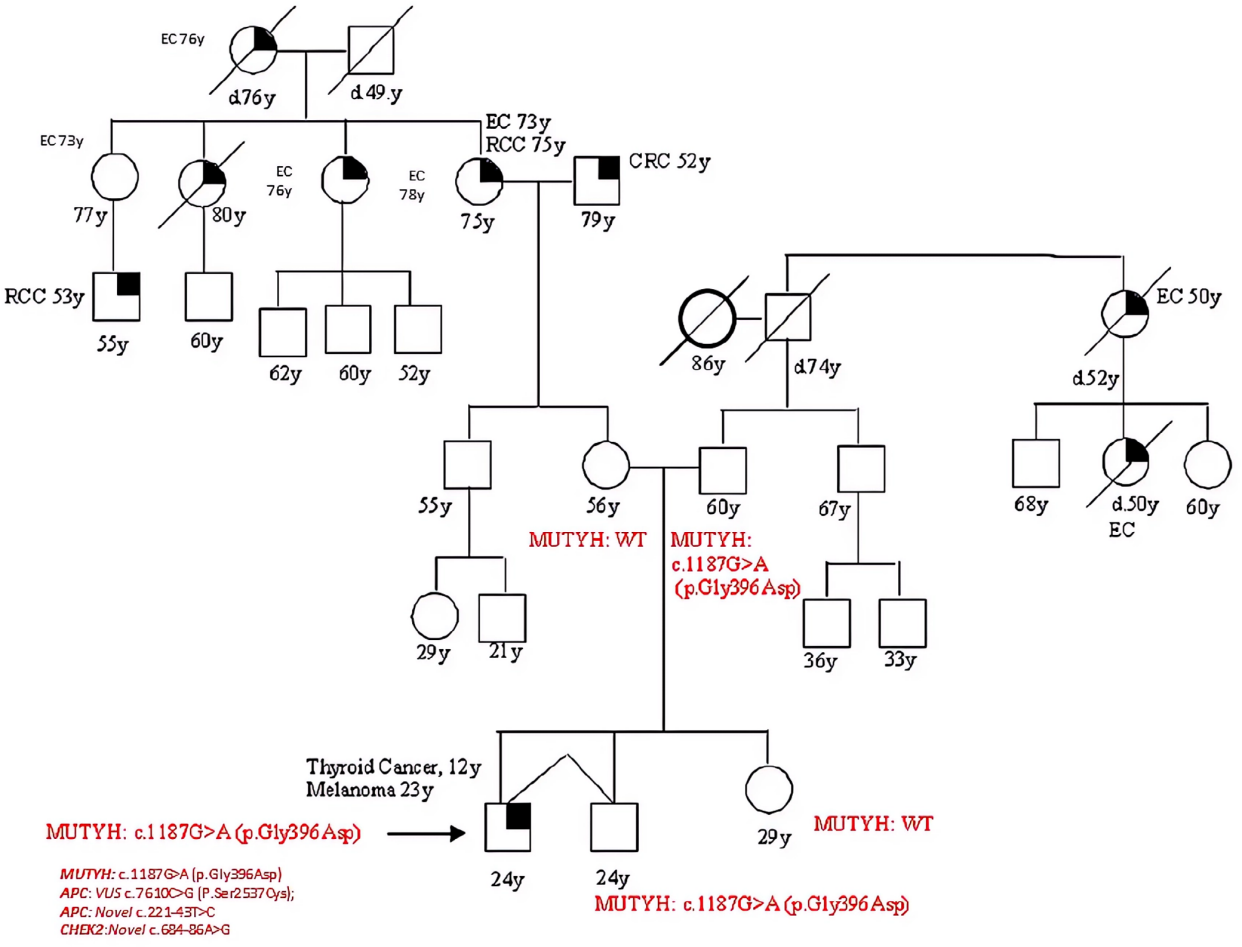
Pedigree of the proband carrying the monoallelic *MUTYH* mutation c.1187G>A (p.Gly396Asp) and novel variants in *APC* and *CHEK2*. CRC, Colorectal cancer; MAP, MUTYH‐associated polyposis; OC, Ovarian cancer; BC, Breast cancer; BBC, Bilateral breast cancer; PC, Prostate cancer; LC, Lung cancer; RCC, Renal cell cancer; GC, Gastric cancer; EC, Endometrial cancer; PaC, Pancreas cancer; TC, Thyroid cancer; TeC, Testicular cancer.

Patient 9 was affected by thyroid cancer and melanoma, diagnosed at 12 and 23 respectively. He had the *MUTYH* mutation c.1187G>A (p.Gly396Asp) and a VUS in *APC* c.7610C>G (p.Ser2537Cys), that was classified as likely pathogenic (class II, PP3) at the bioinformaic analysis (Table 2). This proband carried also 2 novel variants that we describe for the first time: the variant c.221‐43T>C in *APC* and the variant c.684‐86A>G in *CHEK2;* both them resulted likely benign (class IV, BP4) at in silico analysis (Table 2). Patients maternal grandfather had CRC at 52. His identical twin and his father also underwent genetic testing, both turned out to carry the same *MUTYH* mutation while his mother and his sister were wild type (Figure 9).

**FIGURE 10:**
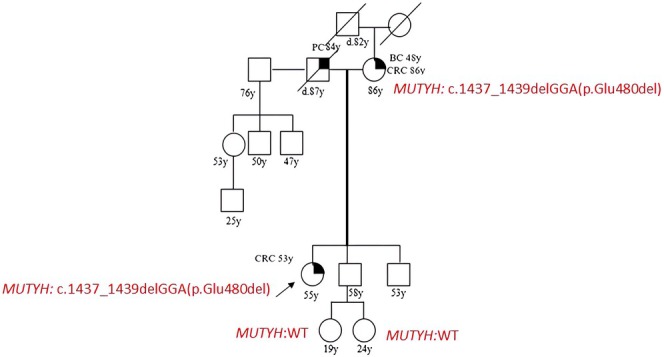
Pedigree of the proband carrying the *MUTYH* mutation c.1437_1439delGGA (p.Glu480del). CRC, Colorectal cancer; MAP, MUTYH‐associated polyposis; OC, Ovarian cancer; BC, Breast cancer; BBC, Bilateral breast cancer; PC, Prostate cancer; LC, Lung cancer; RCC, Renal cell cancer; GC, Gastric cancer; EC, Endometrial cancer; PaC, Pancreas cancer; TC, Thyroid cancer; TeC, Testicular cancer.

Finally patient 10 had CRC at 53 years old, shehad a pathogenic mutation in *MUTYH*, c.1437_1439delGGA (p.Glu480del); at the segregation analysis her mother, who had BC at 48 and CRC at 86, was found to harbor the same mutation. We also tested her brother, who carried the mutation, and her nephews, who were negative (Figure 10).

The last three patients, 11, 12 and 13 had monoallelic variants in *MUTYH* in absence of mutations in other genes included in our panels. Segregation analysis for these patients was not feasible.

**FIGURE 11:**
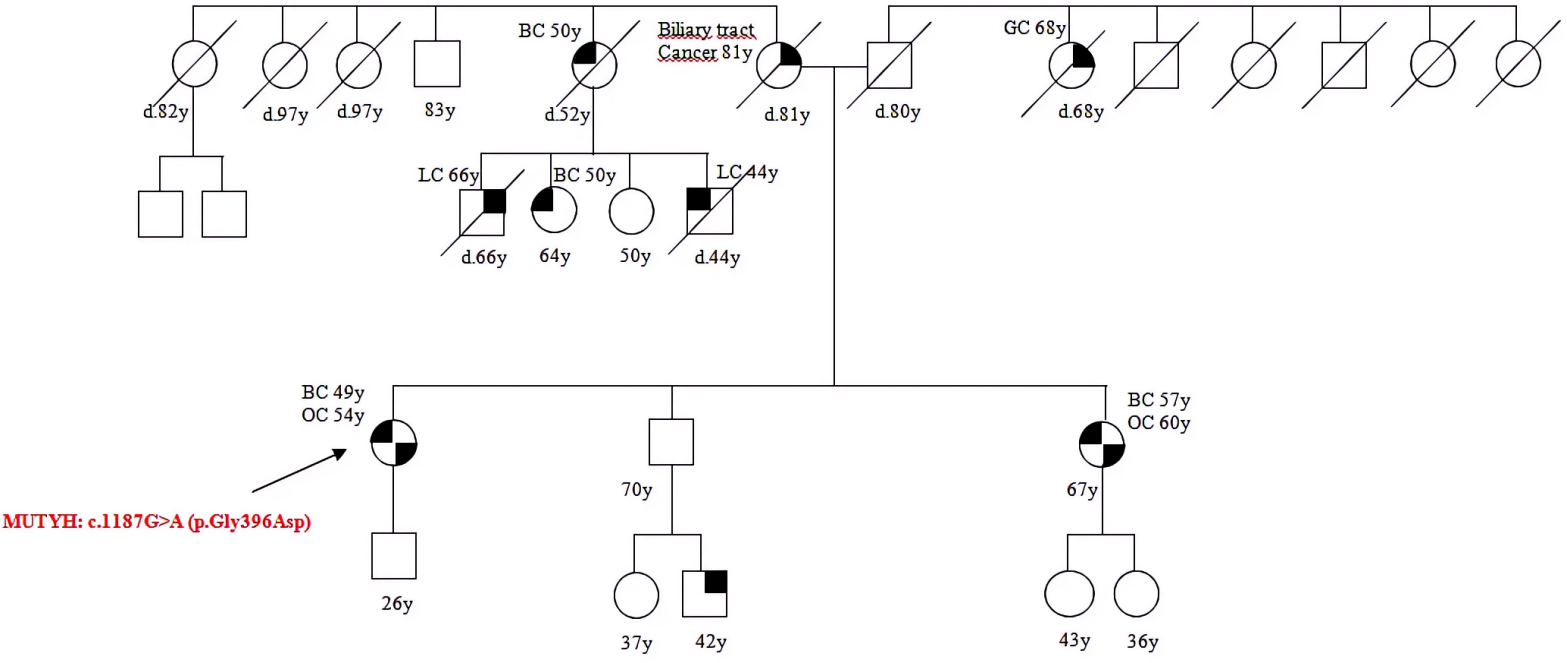
Pedigree of the proband carrying the monoallelic *MUTYH* mutation c.1187G>A (p.Gly396Asp). CRC, Colorectal cancer; MAP, MUTYH‐associated polyposis; OC, Ovarian cancer; BC, Breast cancer; BBC, Bilateral breast cancer; PC, Prostate cancer; LC, Lung cancer; RCC, Renal cell cancer; GC, Gastric cancer; EC, Endometrial cancer; PaC, Pancreas cancer; TC, Thyroid cancer; TeC, Testicular cancer.

Patient 11 presented the *MUTYH* PV c.1187G>A (p.Gly396Asp) and was diagnosed with BC at 49 and OC at 54 (Figure 11).

**FIGURE 12:**
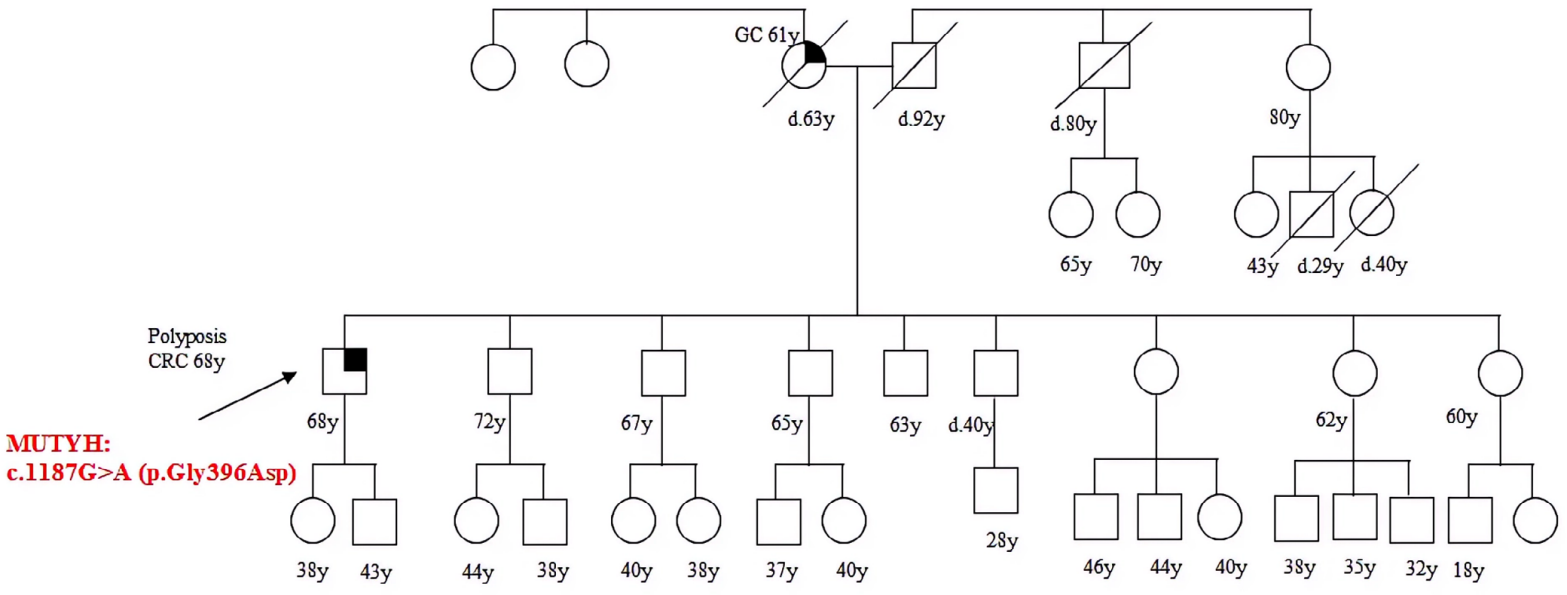
Pedigree of the proband carrying the monoallelic *MUTYH* mutation c.1187G>A (p.Gly396Asp). CRC, Colorectal cancer; MAP, MUTYH‐associated polyposis; OC, Ovarian cancer; BC, Breast cancer; BBC, Bilateral breast cancer; PC, Prostate cancer; LC, Lung cancer; RCC, Renal cell cancer; GC, Gastric cancer; EC, Endometrial cancer; PaC, Pancreas cancer; TC, Thyroid cancer; TeC, Testicular cancer.

Patient 12 carried the *MUTYH* PV c.1187G>A (p.Gly396Asp) and was affected by polyposis and CRC at 68 (Figure 12).

**FIGURE 13:**
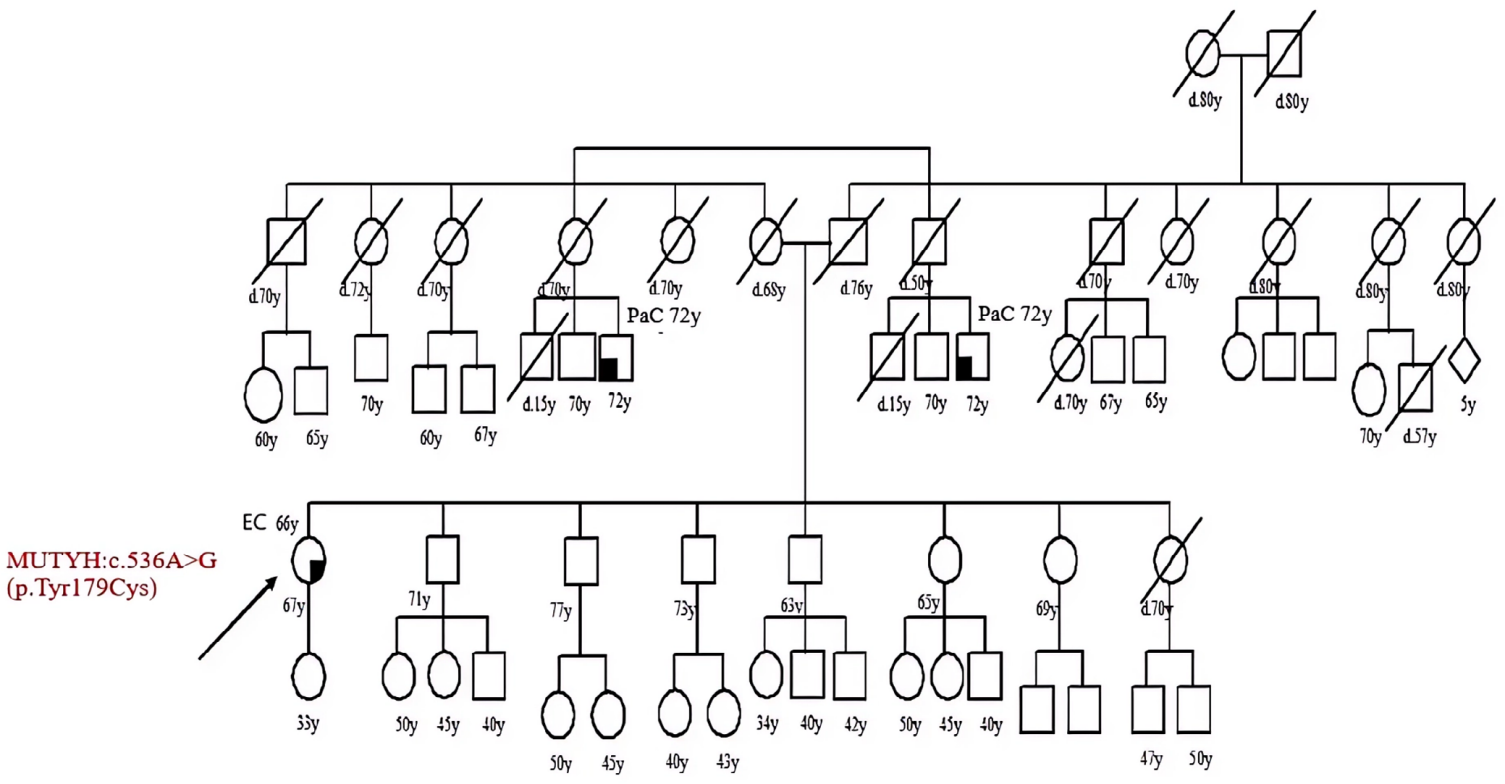
Pedigree of the proband carrying the monoallelic *MUTYH* mutation c.536A>G (p.Tyr179cys). CRC, Colorectal cancer; MAP, MUTYH‐associated polyposis; OC, Ovarian cancer; BC, Breast cancer; BBC, Bilateral breast cancer; PC, Prostate cancer; LC, Lung cancer; RCC, Renal cell cancer; GC, Gastric cancer; EC, Endometrial cancer; PaC, Pancreas cancer; TC, Thyroid cancer; TeC, Testicular cancer.

Patient 13 suffered from MSI endometrial cancer at 66 and her genetic test showed the *MUTYH* mutation c.536A>G (p.Tyr179cys), she denied family history of cancer but her cousin had pancreas cancer at 72 (Figure 13).

**FIGURE 14:**
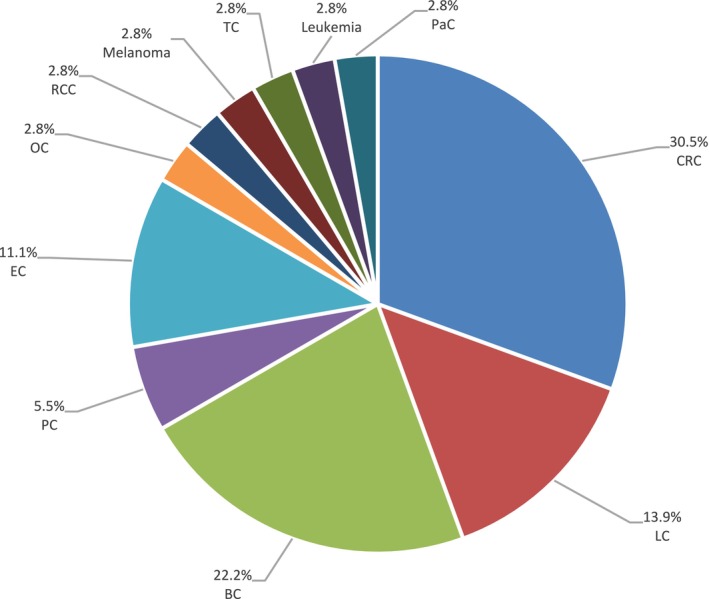
Different cancer types among patients carrying monoallelic MUTYH variants. BC, Breast cancer; CRC, Colorectal cancer; EC, Endometrial cancer; LC, Lung cancer; OC, Ovarian cancer; PaC, Pancreatic cancer; PC, Prostate cancer; RCC, Renal cell cancer; TC, Thyroid cancer.

Figure 14 illustrates the distribution of tumor types observed among patients and their relatives. For this analysis, we considered the family side from which the proband inherited the *MUTYH* mutation and included all tumors that occurred in relatives. We excluded probands with biallelic *MUTYH* mutations or co‐occurring PVs in other cancer susceptibility genes. Overall, we observed a high frequency of CRC (30.5%), followed by BC (22.2%), lung cancer (13.9%) and endometrial cancer (11.1%).

## 4 Discussion

Our findings are interesting for multiple aspects: the first one is the high rate of *MUTYH* mutations observed in our cancer cohort, in particular the missense PVs c.1187G>A (p.Gly396Asp) and c.536A>G (p.Tyr179cys); the second one is the association between monoallelic MUTYH mutation and cancer, suggested by the different mutation rate observed in the cancer cohort compared with the control group and supported by the relationship between the *MUTYH* mutation and cancer through the segregation and co‐segregation analysis.

In regards to the first point, we found a *MUTYH* mutation rate of 10% and the missense mutations c.1187G>A (p.Gly396Asp) and c.536A>G (p.Tyr179cys), recurring in 62% and 31% of the mutated probands, were the most frequent with a cumulative prevalence of 9.2% (6.2% and 3% respectively). Such data are consistent with published literature as we enrolled a highly selected population, made of patients referred for suspicious heredo‐familial cancer and with cases of CRC, polyposis, and other cancer in their pedigree. Our 10% prevalence rate is in the middle between the one observed in individuals with polyposis and the one detected in patients with sporadic CRC. Screening of large series has shown that the combined p.Tyr179Cys and p.Gly396Asp allele frequency in healthy controls of European origin ranges from 0.2% to 1.14% [27]; Webb et al. [7], considering their work and a series of previously published papers, reported a prevalence rate for the common PVs c.1187G>A (p.Gly396Asp) and c.536A>G (p.Tyr179cys), in patients with CRC, ranging from 0.5 [28] to 2.6 [29]; a much higher frequency, varying from 2.2% to 16.8%, has been observed in individuals with polyposis [27].

We also identified the *MUTYH* variant c.1437_1439delGGA (p.Glu480del) in a patient (case 10 in Table 1, Figure 10). This in‐frame deletion of three base pairs results in the loss of a single glutamic acid in the *MUTYH* protein and is well known to be pathogenic when present in a homozygous state or in combination with another pathogenic *MUTYH* variant. In our study, this variant was associated with CRC in both the proband and her mother, from whom she inherited the genetic alteration, despite being monoallelic. A similar case was previously described by Dell'Elice et al., who reported an individual with heterozygous c.1437_1439delGGA (p.Glu480del) and polyposis [30].

The second point, the relationship between *MUTYH* monoallelic variants and cancer predisposition, is much more controversial: three different studies so far concluded with a slightly increased risk of CRC in monoallelic carriers of *MUTYH* PVs [3, 4, 5] though other researchers do not agree [7, 31]. More recently, several research groups explored the hypothesis that *MUTYH* may be implicated in tumors other than CRC, making the scenario even more complex.

Rizzolo and his group [32] suggested a link between *MUTYH* and male BC: they compared 500 cases of *BRCA*‐negative male BC with 1540 controls; it was found a *MUTYH* mutation rate (monoallelic) of 2.5% in the patients' group and 1.3% in the controls group. Such difference resulted statistically significant at the multivariate analysis—OR = 4.54, suggesting a role for monoallelic *MUTYH* mutations in male BC risk. In 2022, Barreiro et al. [33] proposed that somatic LOH (Loss of Heterozigosity) in individuals who are carriers of pathogenic, monoallelic variants of *MUTYH* may represent a mechanism of tumorigenesis. They compared the prevalence of pathogenic monoallelic *MUTYH* variants between a large cancer cohort (from The Cancer Genome Atlas, TCGA) and an equally large cohort of healthy individuals (from Genome Aggregation Database, gnomAD). The reported prevalence was 1.44% in the healthy population and 2.1% in the cancer population, suggesting a role of germline monoallelic *MUTYH* variants in cancer development; a high frequency of monoallelic MUTYH variants was found in adrenocortical carcinoma (ACC), esophageal carcinoma, and sarcoma. Lintas et al. [16] examined 9 cases of familial gynecologic cancer (breast, endometrial, ovarian) with monoallelic variants of *MUTYH*, comparing their germline *MUTYH* status with the somatic variants present in tumor tissue. Though the authors failed to demonstrate LOH in tumor tissue, which would have corroborated the hypothesis of LOH as a mechanism of tumorigenesis, they found absent or reduced expression of *MUTYH* in immunohistochemistry and suggested that dysregulation of the gene may be implicated in carcinogenesis through different mechanisms other than LOH.

Additional studies support a broader contribution of *MUTYH* dysfunction beyond CRC. In one study investigating the relationship between breast and ovarian cancer predisposition and germline and somatic variants of *MUTYH*, *OGG1*, and *BRCA1*, 18 variants (including four novel ones) were identified, and a strong correlation between *MUTYH* and *OGG1* expression was observed in tumor tissue but not in controls, suggesting coordinated BER pathway dysregulation [34]. Furthermore, it has been reported that impaired *MUTYH* function, through disruption of the BER pathway, may lead to oxidative DNA damage and G:C>T:A transversions in multiple tissues, thereby facilitating oncogenesis beyond the colon [35].

The high frequency of BC and endometrial cancers observed in our patients is in line with the findings of these studies, further supporting the hypothesis that MUTYH dysfunction may contribute to tumorigenesis at extra‐colon sites.

On the other hand, it is worth underlining that experts opinions are not unanimous, two recently published papers denied the link between *MUTYH* PVs and increased cancer risk: Thompson et al. [36] found a 1.8% rate of *MUTYH* monoallelic variants in a huge cancer cohort composed of CRC, BC, and endometrial cancer and concluded that there was no significant difference in the prevalence of monoallelic *MUTYH* pathogenic variants among cancer patients compared to controls. Equally, Pena‐Lopez et al. [37] published a retrospective study focused on patients assessed in the hereditary cancer unit in Madrid; they found *MUTYH* variants in 56 cases (rate: 1.6%); the most frequent mutations were c.1187G>A (p.Gly396Asp), 62.9% and c.536A>G (p.Tyr179cys), 9.7%; 89.2% had heterozygous mutations, while the other 6 had homozygous *MUTYH* mutations (*n =* 2) or a monoallelic mutation in *MUTYH* plus a pathogenic/likely pathogenic mutation in another gene (*n =* 4); once again, it was concluded that there was no relationship between BC risk and monoallelic *MUTYH* variants based on their data.

Looking at our results, harboring a *MUTYH* mutation, even if monoallelic, influences the clinical phenotype. This is suggested by the different *MUTYH* mutation rate among the two groups (cancer cohort vs. controls) but also by the results of the segregation analysis; in particular, for the two patients with biallelic mutations in MUTYH (patient 1 and patient 2 in Table 1, Figures 1 and 2), we found that their parents both had a family history of cancer even if they were heterozygous carriers of the *MUTYH* PV observed in their descendants. Besides, we tested at least one parent for 2 patients (patient 3 and patient 5 in Table 1, Figures 3 and 5) with DM (*MUTYH* PV and mutation in another cancer gene) and we verified that the former inherited the MUTYH mutation from her father and the *CHEK 2* mutation from her mother, and the latter the *MUTYH* mutation from his mother and the *MLH1* mutation from his father. In these patients, the presence of cancer in individuals from both branches of the family strengthens the hypothesis that the *MUTYH* mutation is pathogenic itself, even when monoallelic.

Regarding the potential additive risk conferred by additional PVs in cancer susceptibility genes beyond a monoallelic *MUTYH* PV, their significance remains largely unexplored. Given the growing interest in BRCA among women with BC, both case reports and case series have recently been published describing the co‐occurrence of PVs in *BRCA1* or *BRCA2* with PVs in another Hereditary Breast and Ovarian Cancer (HBOC) associated gene, as well as the co‐occurrence of PVs in both *BRCA1* and *BRCA2*. The results are controversial, with some authors reporting earlier age at onset and more aggressive phenotypes [38, 39, 40], and synchronous tumors [41], while others have not confirmed these findings [42]. For less‐studied genes such as *MUTYH*, it is even more challenging to determine the effects of concomitant PVs. With the increasing use of multigene panels, data on PV co‐occurrence will grow, enhancing our understanding of additive risk and guiding clinical management.

Our sample size is not large enough to draw definitive conclusions; however, these findings provide supportive evidence for a potential role of monoallelic *MUTYH* in cancer predisposition. Further studies are warranted to elucidate the mechanisms by which such variants may increase cancer risk. Screening for *MUTYH* could be considered in all patients with suspected hereditary cancer, even when they do not meet the traditional criteria for a specific hereditary cancer syndrome. A careful evaluation of family history is also essential to identify patients with coexisting cancer types (e.g., BC and CRC, endometrial cancer and CRC), who may benefit from testing with expanded hereditary cancer panels. Moreover, clarifying the role of monoallelic *MUTYH* PVs in tumor development could inform targeted cancer prevention strategies for healthy carriers.

## Author Contributions

**Gemma Caliendo:** conceptualization, investigation, validation, data curation, supervision. **Chiara Della Pepa:** writing – review and editing, writing – original draft, data curation, investigation. **Alessia Mignano:** writing – original draft, methodology, formal analysis, software, data curation. **Luisa Albanese:** methodology, formal analysis. **Luana Passariello:** methodology, formal analysis. **Anna Cozzolino:** methodology, formal analysis. **Francesca Iengo:** methodology, formal analysis. **Anna Maria Molinari:** writing – review and editing, supervision. **Laura Pesce:** writing – review and editing, data curation. **Maria Teresa Vietri:** conceptualization, writing – review and editing, supervision.

## Ethics Statement

This study was carried out in accordance with the World Medical Association Helsinki Declaration. Informed consent was obtained from all the subjects, and the study was approved by the ethics committee of the University of Campania “Luigi Vanvitelli” and conducted according to the ethical guidelines of the University of Campania “Luigi Vanvitelli” (n.25455‐07/09/2021).

## Consent

Informed consent was obtained from all subjects involved in the study.

## Conflicts of Interest

The authors declare no conflicts of interest.

## Data Availability

The data generated in this research are entirely described in the manuscript.
